# Butane-1,4-diaminium 2-(meth­oxy­carbon­yl)benzoate dihydrate

**DOI:** 10.1107/S1600536811003618

**Published:** 2011-02-09

**Authors:** Jian Li

**Affiliations:** a Department of Chemistry and Chemical Engineering, Weifang University, Weifang 261061, People’s Republic of China

## Abstract

In the title compound, C_4_H_14_N_2_^+^·2C_9_H_7_O_4_^−^·2H_2_O, the butane-1,4-diaminium cation lies on an inversion center. In the crystal, inter­molecular N—H⋯O and O—H⋯O hydrogen bonds link the components into layers parallel to (100). Addtional stabilization within these layers is provided by weak inter­molecular C—H⋯O hydrogen bonds.

## Related literature

For the appications of phthalimides and *N*-substituted phthalimides, see: Lima *et al.* (2002 ▶). For a related structure, see: Liang (2008 ▶).
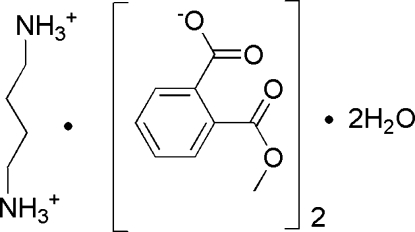


## Experimental

### 

#### Crystal data

C_4_H_14_N_2_^2+^·2C_9_H_7_O_4_^−^·2H_2_O
*M* *_r_* = 484.50
Monoclinic, 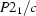

*a* = 14.0344 (15) Å
*b* = 8.6746 (9) Å
*c* = 10.2304 (11) Å
β = 95.620 (1)°
*V* = 1239.5 (2) Å^3^
*Z* = 2
Mo *K*α radiation
μ = 0.10 mm^−1^
*T* = 298 K
0.50 × 0.48 × 0.47 mm

#### Data collection

Bruker SMART CCD diffractometer
Absorption correction: multi-scan (*SADABS*; Bruker, 1997 ▶) *T*_min_ = 0.950, *T*_max_ = 0.953
6001 measured reflections
2178 independent reflections
1601 reflections with *I* > 2σ(*I*)
*R*_int_ = 0.037

#### Refinement

*R*[*F* ^2^ > 2σ(*F* ^2^)] = 0.043
*wR*(*F* ^2^) = 0.123
*S* = 1.07
2178 reflections
157 parameters
H-atom parameters constrained
Δρ_max_ = 0.17 e Å^−3^
Δρ_min_ = −0.20 e Å^−3^

### 

Data collection: *SMART* (Bruker, 1997 ▶); cell refinement: *SAINT* (Bruker, 1997 ▶); data reduction: *SAINT*; program(s) used to solve structure: *SHELXS97* (Sheldrick, 2008 ▶); program(s) used to refine structure: *SHELXL97* (Sheldrick, 2008 ▶); molecular graphics: *SHELXTL* (Sheldrick, 2008 ▶) and *PLATON* (Spek, 2009 ▶); software used to prepare material for publication: *SHELXTL*.

## Supplementary Material

Crystal structure: contains datablocks global, I. DOI: 10.1107/S1600536811003618/lh5202sup1.cif

Structure factors: contains datablocks I. DOI: 10.1107/S1600536811003618/lh5202Isup2.hkl

Additional supplementary materials:  crystallographic information; 3D view; checkCIF report

## Figures and Tables

**Table 1:** Hydrogen-bond geometry (Å, °)

*D*—H⋯*A*	*D*—H	H⋯*A*	*D*⋯*A*	*D*—H⋯*A*
N1—H1*A*⋯O4^i^	0.89	1.95	2.815 (2)	164
N1—H1*B*⋯O3	0.89	2.00	2.823 (2)	154
N1—H1*C*⋯O5	0.89	1.99	2.876 (2)	172
O5—H5*C*⋯O3^ii^	0.85	2.03	2.873 (2)	172
O5—H5*D*⋯O4^iii^	0.85	1.96	2.808 (2)	172
C11—H11*A*⋯O2^i^	0.97	2.46	3.346 (2)	151

Symmetry codes: (i) 
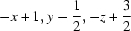
; (ii) 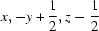; (iii) 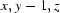.

**Fig. 1.**
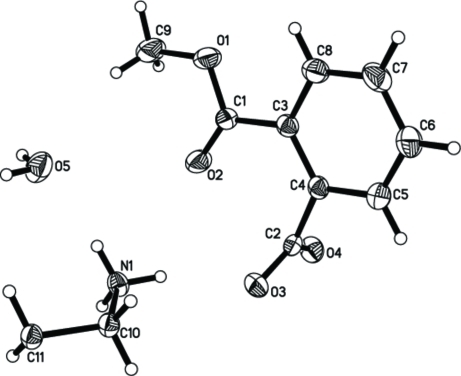
The asymmetric unit of (I), drawn with 30% probability ellipsoids.

**Fig. 2.**
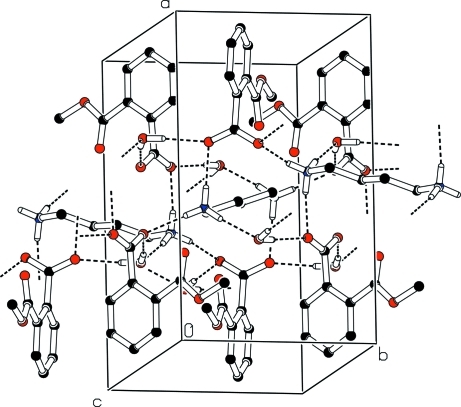
Part of the crystal structure of the title compound with hydrogen bonds shown as dashed lines. Only H atoms involved in hydrogen bonds are shown.

## supplementary crystallographic information

### Comment

Phthalimides and N-substituted phthalimides are animportant class of compounds because of their interesting biological activities (Lima *et al.*, 2002). 2-(Methoxycarbonyl)benzoic acid is an intermediate in the preparation of N-substituted phthalimides. In this paper, the structure of the title compound is reported. The asymmetric unit of the title compound (I) contains one half a butane-1,4-diaminium cation, a 2-(methoxycarbonyl)benzoate anion and a solvent water molecule (Fig. 1). The bond lengths and angles agree with those in ethane-1,2-diaminium 2-(methoxycarbonyl)-3,4,5,6-tetrabromobenzoate methanol solvate (Liang, 2008). In the crystal, intermolecular N—H···O and O—H···O hydrogen bonds link the components of the structure into two-dimensional layers parallel to (100) (Fig. 2 and Table 1). Addtional stabilization within these layers is provided by weak intermolecular C—H···O hydrogen bonds.

### Experimental

A mixture of phthalic anhydride (1.52 g, 0.01 mol) and methanol (15 ml) was refluxed for 0.5 h. 1,4-Butanediamine (0.44 g, 0.005 mol) was added to the above solution and mixed for 10 min at room temperature. The solution was kept at room temperature for 5 d. Natural evaporation gave colourless single crystals of the title compound, suitable for X-ray analysis.

### Refinement

H atoms were initially located in difference maps and then refined in a riding-model approximation with C—H = 0.93–0.97 Å, N—H = 0.89 Å, O—H = 0.82Å and *U*_iso_(H) = 1.2*U*_eq_(C, O) or 1.5*U*_eq_(N, methyl C).

### Crystal data

**Table:**

C_4_H_14_N_2_^2+^·2C_9_H_7_O_4_^−^·2H_2_O	*F*(000) = 516
*M**_r_* = 484.50	*D*_x_ = 1.298 Mg m^−^^3^
Monoclinic, *P*2_1_/*c*	Mo *K*α radiation, λ = 0.71073 Å
Hall symbol: -P 2ybc	Cell parameters from 2198 reflections
*a* = 14.0344 (15) Å	θ = 2.8–27.5°
*b* = 8.6746 (9) Å	µ = 0.10 mm^−^^1^
*c* = 10.2304 (11) Å	*T* = 298 K
β = 95.620 (1)°	Block, colorless
*V* = 1239.5 (2) Å^3^	0.50 × 0.48 × 0.47 mm
*Z* = 2	

### Data collection

**Table:**

Bruker SMART CCD diffractometer	2178 independent reflections
Radiation source: fine-focus sealed tube	1601 reflections with *I* > 2σ(*I*)
graphite	*R*_int_ = 0.037
φ and ω scans	θ_max_ = 25.0°, θ_min_ = 2.8°
Absorption correction: multi-scan (*SADABS*; Bruker, 1997)	*h* = −12→16
*T*_min_ = 0.950, *T*_max_ = 0.953	*k* = −10→10
6001 measured reflections	*l* = −10→12

### Refinement

**Table:**

Refinement on *F*^2^	Secondary atom site location: difference Fourier map
Least-squares matrix: full	Hydrogen site location: inferred from neighbouring sites
*R*[*F*^2^ > 2σ(*F*^2^)] = 0.043	H-atom parameters constrained
*wR*(*F*^2^) = 0.123	*w* = 1/[σ^2^(*F*_o_^2^) + (0.0556*P*)^2^ + 0.314*P*] where *P* = (*F*_o_^2^ + 2*F*_c_^2^)/3
*S* = 1.07	(Δ/σ)_max_ < 0.001
2178 reflections	Δρ_max_ = 0.17 e Å^−^^3^
157 parameters	Δρ_min_ = −0.20 e Å^−^^3^
0 restraints	Extinction correction: *SHELXL97* (Sheldrick, 2008), Fc^*^=kFc[1+0.001xFc^2^λ^3^/sin(2θ)]^-1/4^
Primary atom site location: structure-invariant direct methods	Extinction coefficient: 0.118 (8)

### Special details

**Table:**

Geometry. All e.s.d.'s (except the e.s.d. in the dihedral angle between two l.s. planes) are estimated using the full covariance matrix. The cell e.s.d.'s are taken into account individually in the estimation of e.s.d.'s in distances, angles and torsion angles; correlations between e.s.d.'s in cell parameters are only used when they are defined by crystal symmetry. An approximate (isotropic) treatment of cell e.s.d.'s is used for estimating e.s.d.'s involving l.s. planes.
Refinement. Refinement of *F*^2^ against ALL reflections. The weighted *R*-factor *wR* and goodness of fit *S* are based on *F*^2^, conventional *R*-factors *R* are based on *F*, with *F* set to zero for negative *F*^2^. The threshold expression of *F*^2^ > σ(*F*^2^) is used only for calculating *R*-factors(gt) *etc*. and is not relevant to the choice of reflections for refinement. *R*-factors based on *F*^2^ are statistically about twice as large as those based on *F*, and *R*- factors based on ALL data will be even larger.

### Fractional atomic coordinates and isotropic or equivalent isotropic displacement parameters (Å^2^)

**Table:**

	*x*	*y*	*z*	*U*_iso_*/*U*_eq_	
N1	0.44862 (11)	0.25834 (17)	0.79462 (15)	0.0369 (4)	
H1A	0.5061	0.2597	0.7647	0.055*	
H1B	0.4252	0.3537	0.7944	0.055*	
H1C	0.4094	0.1986	0.7432	0.055*	
O1	0.13265 (10)	0.39089 (19)	0.54038 (16)	0.0622 (5)	
O2	0.28346 (10)	0.43510 (19)	0.61583 (16)	0.0596 (5)	
O3	0.37929 (10)	0.54277 (15)	0.88018 (14)	0.0458 (4)	
O4	0.35667 (9)	0.74614 (15)	0.74950 (14)	0.0479 (4)	
O5	0.33244 (12)	0.04093 (17)	0.63792 (15)	0.0628 (5)	
H5C	0.3453	0.0256	0.5595	0.075*	
H5D	0.3382	−0.0443	0.6790	0.075*	
C1	0.19982 (14)	0.4525 (2)	0.62583 (19)	0.0376 (5)	
C2	0.32751 (13)	0.6373 (2)	0.81441 (18)	0.0342 (4)	
C3	0.15919 (12)	0.5400 (2)	0.73169 (18)	0.0351 (5)	
C4	0.22064 (13)	0.6245 (2)	0.82091 (18)	0.0344 (5)	
C5	0.18168 (15)	0.7007 (2)	0.9231 (2)	0.0490 (6)	
H5	0.2216	0.7570	0.9833	0.059*	
C6	0.08535 (16)	0.6941 (3)	0.9366 (2)	0.0587 (6)	
H6	0.0608	0.7457	1.0056	0.070*	
C7	0.02511 (16)	0.6117 (3)	0.8485 (2)	0.0565 (6)	
H7	−0.0401	0.6075	0.8579	0.068*	
C8	0.06155 (14)	0.5354 (2)	0.7462 (2)	0.0469 (5)	
H8	0.0206	0.4804	0.6863	0.056*	
C9	0.16701 (18)	0.2992 (4)	0.4371 (3)	0.0783 (9)	
H9A	0.2048	0.3625	0.3851	0.117*	
H9B	0.1135	0.2580	0.3825	0.117*	
H9C	0.2055	0.2161	0.4751	0.117*	
C10	0.45726 (13)	0.1967 (2)	0.93045 (17)	0.0337 (4)	
H10A	0.3955	0.2014	0.9654	0.040*	
H10B	0.5020	0.2592	0.9860	0.040*	
C11	0.49175 (14)	0.0324 (2)	0.93138 (17)	0.0353 (5)	
H11A	0.5510	0.0274	0.8901	0.042*	
H11B	0.4447	−0.0306	0.8801	0.042*	

### Atomic displacement parameters (Å^2^)

**Table:**

	*U*^11^	*U*^22^	*U*^33^	*U*^12^	*U*^13^	*U*^23^
N1	0.0428 (9)	0.0320 (8)	0.0361 (9)	0.0064 (7)	0.0048 (7)	0.0075 (7)
O1	0.0412 (8)	0.0820 (12)	0.0614 (11)	−0.0001 (8)	−0.0048 (7)	−0.0318 (9)
O2	0.0391 (9)	0.0752 (11)	0.0644 (11)	−0.0021 (7)	0.0052 (7)	−0.0322 (9)
O3	0.0441 (8)	0.0439 (8)	0.0481 (9)	0.0115 (6)	−0.0026 (6)	0.0025 (7)
O4	0.0462 (9)	0.0374 (8)	0.0612 (10)	−0.0023 (6)	0.0106 (7)	0.0063 (7)
O5	0.0936 (13)	0.0428 (8)	0.0488 (10)	−0.0037 (8)	−0.0094 (8)	0.0011 (7)
C1	0.0365 (11)	0.0357 (10)	0.0398 (11)	−0.0011 (8)	0.0001 (8)	−0.0013 (8)
C2	0.0391 (10)	0.0279 (9)	0.0351 (10)	0.0004 (8)	0.0008 (8)	−0.0071 (8)
C3	0.0352 (10)	0.0325 (10)	0.0374 (11)	0.0026 (8)	0.0023 (8)	0.0043 (8)
C4	0.0382 (10)	0.0275 (9)	0.0372 (11)	0.0038 (8)	0.0024 (8)	0.0037 (8)
C5	0.0479 (13)	0.0505 (12)	0.0486 (13)	0.0046 (10)	0.0041 (10)	−0.0111 (10)
C6	0.0530 (14)	0.0701 (15)	0.0548 (15)	0.0104 (12)	0.0146 (11)	−0.0149 (12)
C7	0.0386 (12)	0.0694 (15)	0.0634 (15)	0.0079 (11)	0.0144 (10)	−0.0010 (13)
C8	0.0378 (11)	0.0493 (12)	0.0530 (14)	−0.0004 (9)	0.0014 (9)	0.0017 (10)
C9	0.0604 (16)	0.101 (2)	0.0702 (19)	0.0054 (14)	−0.0111 (13)	−0.0481 (16)
C10	0.0425 (11)	0.0302 (9)	0.0286 (10)	0.0033 (8)	0.0044 (8)	0.0020 (8)
C11	0.0457 (11)	0.0307 (9)	0.0295 (10)	0.0041 (8)	0.0035 (8)	0.0007 (8)

### Geometric parameters (Å, °)

**Table:**

N1—C10	1.483 (2)	C5—C6	1.373 (3)
N1—H1A	0.8900	C5—H5	0.9300
N1—H1B	0.8900	C6—C7	1.374 (3)
N1—H1C	0.8900	C6—H6	0.9300
O1—C1	1.333 (2)	C7—C8	1.378 (3)
O1—C9	1.443 (3)	C7—H7	0.9300
O2—C1	1.198 (2)	C8—H8	0.9300
O3—C2	1.248 (2)	C9—H9A	0.9600
O4—C2	1.246 (2)	C9—H9B	0.9600
O5—H5C	0.8500	C9—H9C	0.9600
O5—H5D	0.8500	C10—C11	1.505 (2)
C1—C3	1.481 (3)	C10—H10A	0.9700
C2—C4	1.512 (3)	C10—H10B	0.9700
C3—C8	1.393 (3)	C11—C11^i^	1.509 (3)
C3—C4	1.400 (3)	C11—H11A	0.9700
C4—C5	1.393 (3)	C11—H11B	0.9700
			
C10—N1—H1A	109.5	C7—C6—H6	119.9
C10—N1—H1B	109.5	C6—C7—C8	119.8 (2)
H1A—N1—H1B	109.5	C6—C7—H7	120.1
C10—N1—H1C	109.5	C8—C7—H7	120.1
H1A—N1—H1C	109.5	C7—C8—C3	120.6 (2)
H1B—N1—H1C	109.5	C7—C8—H8	119.7
C1—O1—C9	115.83 (17)	C3—C8—H8	119.7
H5C—O5—H5D	108.2	O1—C9—H9A	109.5
O2—C1—O1	121.97 (18)	O1—C9—H9B	109.5
O2—C1—C3	125.28 (17)	H9A—C9—H9B	109.5
O1—C1—C3	112.75 (16)	O1—C9—H9C	109.5
O4—C2—O3	125.51 (18)	H9A—C9—H9C	109.5
O4—C2—C4	117.28 (16)	H9B—C9—H9C	109.5
O3—C2—C4	117.10 (16)	N1—C10—C11	110.12 (14)
C8—C3—C4	119.66 (18)	N1—C10—H10A	109.6
C8—C3—C1	121.09 (17)	C11—C10—H10A	109.6
C4—C3—C1	119.22 (16)	N1—C10—H10B	109.6
C5—C4—C3	118.40 (17)	C11—C10—H10B	109.6
C5—C4—C2	117.51 (17)	H10A—C10—H10B	108.2
C3—C4—C2	124.08 (16)	C10—C11—C11^i^	112.22 (19)
C6—C5—C4	121.2 (2)	C10—C11—H11A	109.2
C6—C5—H5	119.4	C11^i^—C11—H11A	109.2
C4—C5—H5	119.4	C10—C11—H11B	109.2
C5—C6—C7	120.3 (2)	C11^i^—C11—H11B	109.2
C5—C6—H6	119.9	H11A—C11—H11B	107.9
			
C9—O1—C1—O2	1.4 (3)	O3—C2—C4—C5	86.2 (2)
C9—O1—C1—C3	−177.8 (2)	O4—C2—C4—C3	90.4 (2)
O2—C1—C3—C8	−170.6 (2)	O3—C2—C4—C3	−93.2 (2)
O1—C1—C3—C8	8.6 (3)	C3—C4—C5—C6	−0.2 (3)
O2—C1—C3—C4	7.4 (3)	C2—C4—C5—C6	−179.6 (2)
O1—C1—C3—C4	−173.44 (17)	C4—C5—C6—C7	−0.1 (4)
C8—C3—C4—C5	0.7 (3)	C5—C6—C7—C8	0.0 (4)
C1—C3—C4—C5	−177.31 (17)	C6—C7—C8—C3	0.5 (3)
C8—C3—C4—C2	−179.96 (17)	C4—C3—C8—C7	−0.8 (3)
C1—C3—C4—C2	2.1 (3)	C1—C3—C8—C7	177.12 (19)
O4—C2—C4—C5	−90.2 (2)	N1—C10—C11—C11^i^	176.08 (19)

Symmetry codes: (i) −*x*+1, −*y*, −*z*+2.

### Hydrogen-bond geometry (Å, °)

**Table:**

*D*—H···*A*	*D*—H	H···*A*	*D*···*A*	*D*—H···*A*
N1—H1A···O4^ii^	0.89	1.95	2.815 (2)	164
N1—H1B···O3	0.89	2.00	2.823 (2)	154
N1—H1C···O5	0.89	1.99	2.876 (2)	172
O5—H5C···O3^iii^	0.85	2.03	2.873 (2)	172
O5—H5D···O4^iv^	0.85	1.96	2.808 (2)	172
C11—H11A···O2^ii^	0.97	2.46	3.346 (2)	151

Symmetry codes: (ii) −*x*+1, *y*−1/2, −*z*+3/2; (iii) *x*, −*y*+1/2, *z*−1/2; (iv) *x*, *y*−1, *z*.
